# MoO_3_NPs/ZIF-8 composite material prepared via RCVD for photodegradation of dyes

**DOI:** 10.1016/j.dib.2018.06.120

**Published:** 2018-07-05

**Authors:** Matteo Ciprian, Peng Xu, Somboon Chaemchuen, Rong Tu, Serge Zhuiykov, Philippe M. Heynderickx, Francis Verpoort

**Affiliations:** a State Key Laboratory of Advanced Technology for Materials Synthesis and Processing, Wuhan University of Technology, Wuhan, China; b Center for Environmental and Energy Research (CEER), Ghent University Global Campus, 119 Songdomunhwa-Ro, Yeonsu-Gu, Incheon 406-840, South Korea; c National Research Tomsk Polytechnic University, Lenin Avenue 30, 634050 Tomsk, Russian Federation; d Department of Green Chemistry and Technology (BW24), Faculty of Bioscience Engineering, Ghent University, 753 Coupure Links, Ghent B-9000, Belgium

## Abstract

Toxic wastewaters from the textile industry have made its way into rivers and other waterways, posing a serious health treat on both human and wildlife. Herein, this data set presents the potential use of MoO_3_ nanoparticles supported on ZIF-8 in the photodegradation of a cationic dye molecule. The data presented in this article report a concise description of experimental conditions for the spray-dried ZIF-8 synthesis and subsequent deposition of MoO_3_ nanoparticles via rotary chemical vapor deposition (RCVD). The photodegradation and analysis data reviled that the MoO_3_-NPs@ZIF-8 3 wt% displayed the ability of degrading methylene blue up to 82% and 95% after 180 and 300 min, respectively.

**Specifications Table**

**Table:** 

**Subject area**	*Chemistry, Environmental Sciences and Engineering*
**More specific subject area**	*Photodegradation*
**Type of data**	*Table, image, graph, figure*
**How data was acquired**	– *Dye concentration measurement: Monitored by UV–vis absorption spectroscopy (UV-1800 Shimadzu, Japan)* – *Catalyst stability: Diffraction spectra were acquired using PXRD spectroscopy (Rigaku Ultima III, Japan), the molecular integrity was analyzed by FT-IR spectroscopy (Nicolet 6700, Thermo Scientific) and the morphology was confirmed by Scanning electron microscope (SEM – Phenom, Ted Pella Inc.)* – *Metal content: Monitored by Inductive coupled plasma atomic Emission (ICP-AES, Optima 4300 DV, PerkinElmer Inc.)*
**Data format**	*Analyzed*
**Experimental factors**	*Activation of MoO*_*3*_*-NPs/ZIF-8 prior to the BETs measurements were achieved by evacuating at 180 °C under vacuum for 6 h.*
**Experimental features**	– *Spry-dried ZIF-8 was synthesized as reported in the original article [“Submitted to Microporous and Mesoporous Materials.”] and literature procedure* [1]*. The MoO*_*3*_ *nanoparticles were deposited onto the ZIF-8 powder by rotary chemical vapor deposition to obtain the final composite material.* – *The photocatalysts were mixed with the dye aqueous solution and monitored for their photodegradation capacity.*
**Data source location**	*Wuhan, P.R. China*
**Data accessibility**	*Data are accessible with article*
**Related research article**	*Matteo Ciprian, Peng Xu, Somboon Chaemchuen, Rong Tu, Serge Zhuiykov, Philippe Heynderickx, Francis Verpoort, MoO*_*3*_*nanoparticle formation on zeolitic imidazolate frameworks-8 by rotary chemical vapor deposition “Submitted to Microporous and Mesoporous Materials.”*

**Value of the data**

• The use of the rotary chemical vapor deposition can encourage researchers to efficiently deposit other compounds on metal-organic frameworks.
• The as-synthesized MoO_3_-NPs/ZIF-8 exhibits good photodegradation properties towards the removal of dye from polluted environment.
• To understand the relationship between an alternative nanoparticle deposition technique and the subsequent properties of the composite material.

## 1 Data

**Fig. 1:**
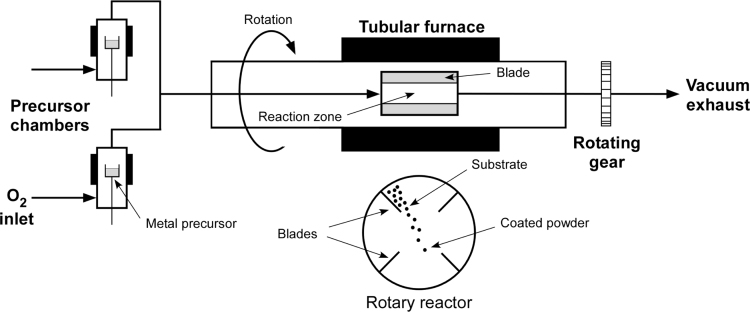
Schematic of RCVD equipment.

**Fig. 2:**
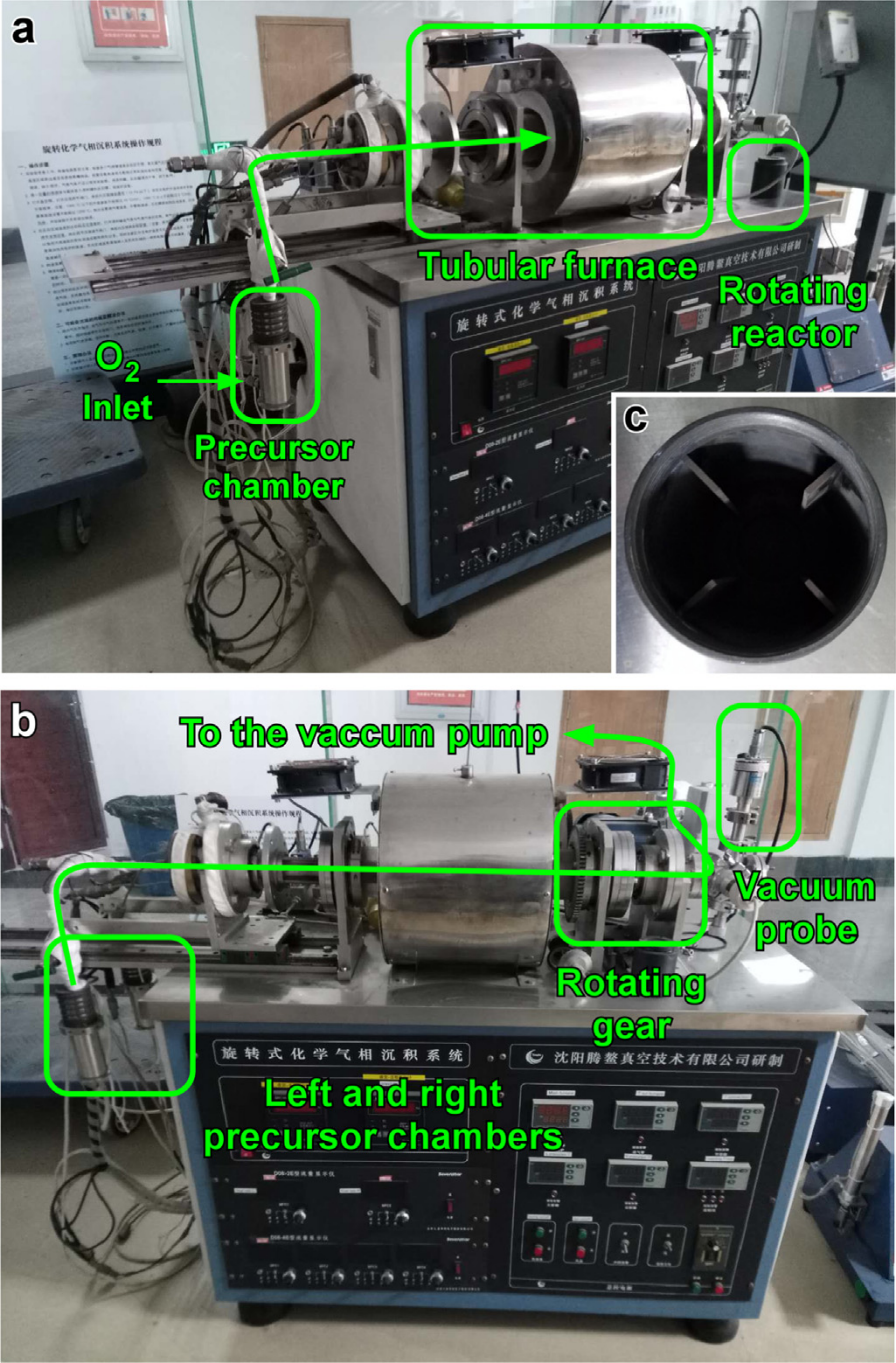
Pictures of the RCVD apparatus, (a) highlight the precursor chamber with the O_2_ inlet, the tubular furnace and the rotating reactor (detail in c). In section (b) the rotating gear and the vacuum probe are displayed.

Nanoparticles deposition and coating of powders have been accomplished with numerous techniques including chemical vapor deposition (CVD) [2] and sol-gel [3]. The combination of a fluidized bed and CVD has been extensively used for coating powders. However, operational limits are imposed due to particle size and density of the powders. Rotary chemical vapor deposition (RCVD) is an alternative method that allows us to remove previous operational restrictions and achieve a uniform nanoparticle deposition [4]. A schematic representation and pictures of the apparatus are reported in Fig. 1, Fig. 2.

**Fig. 3:**
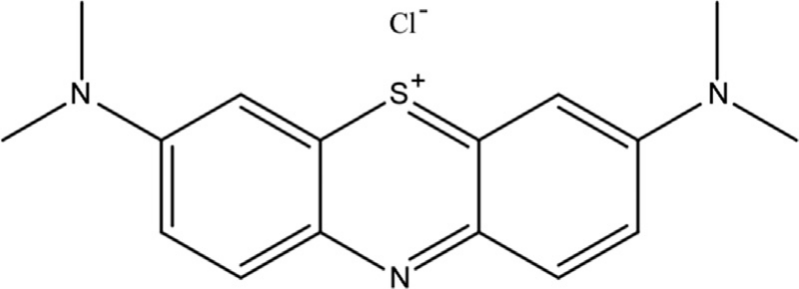
Chemical structure of cationic dye methylene blue.

**Fig. 4:**
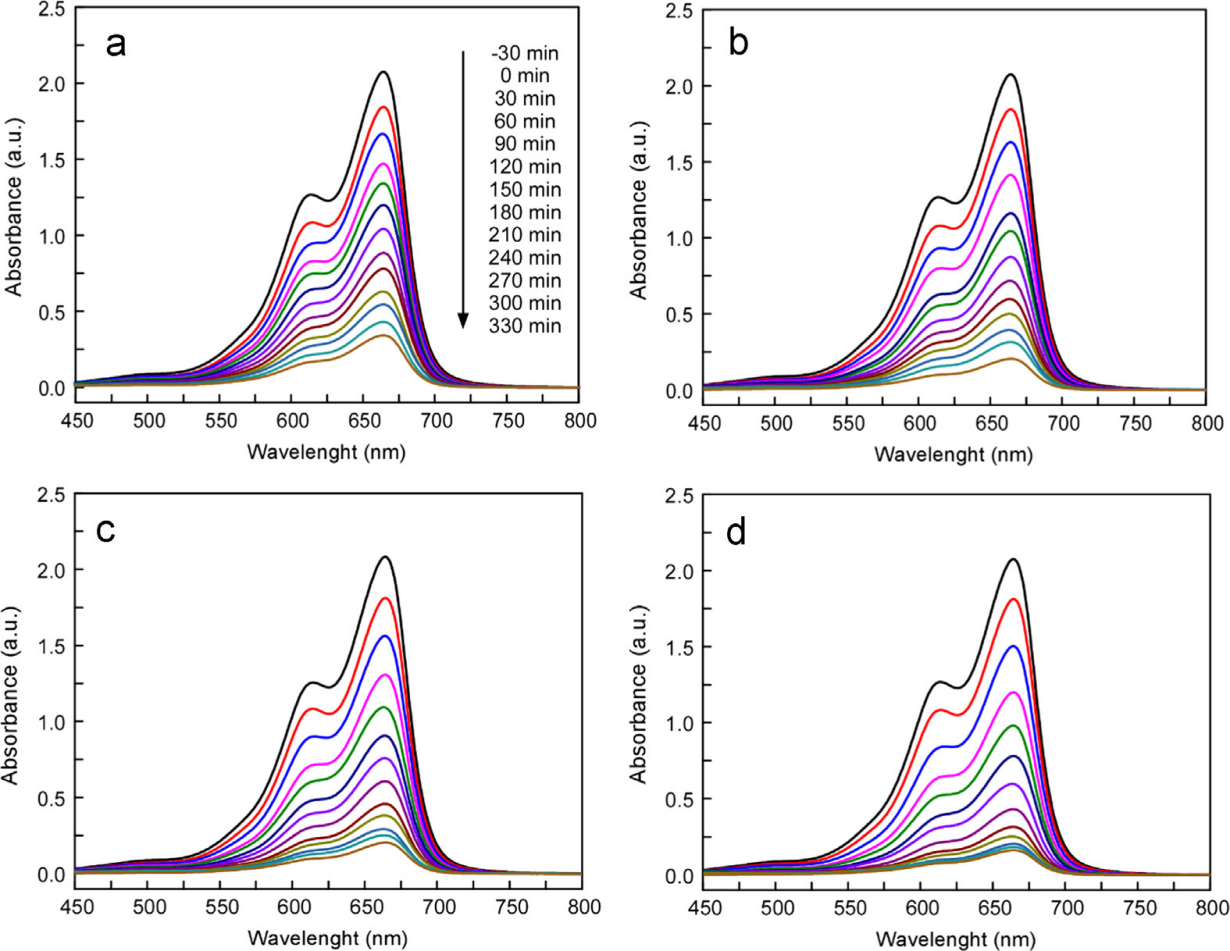
Spectral evaluation of MB degradation using ZIF-8 (a), MoO_3_-NPs/ZIF-8 1, 2 and 3 wt% (b,c,d).

Due to the harmful effects of industrial dye emission to the environment and human health [5], [6], the following data set demonstrate the photocatalytic potential of the as-synthesized MoO_3_-NPs/ZIF-8 on a cationic dye molecule. In Fig. 2 is shown the structure of the methylene blue (MB). A change in maximum absorbance at 664 nm was used to monitor the dye׳s degradation [7]. The UV–vis spectra for the methylene blue photodegradation are illustrated in Fig. 3 (Fig. 4).

**Fig. 5:**
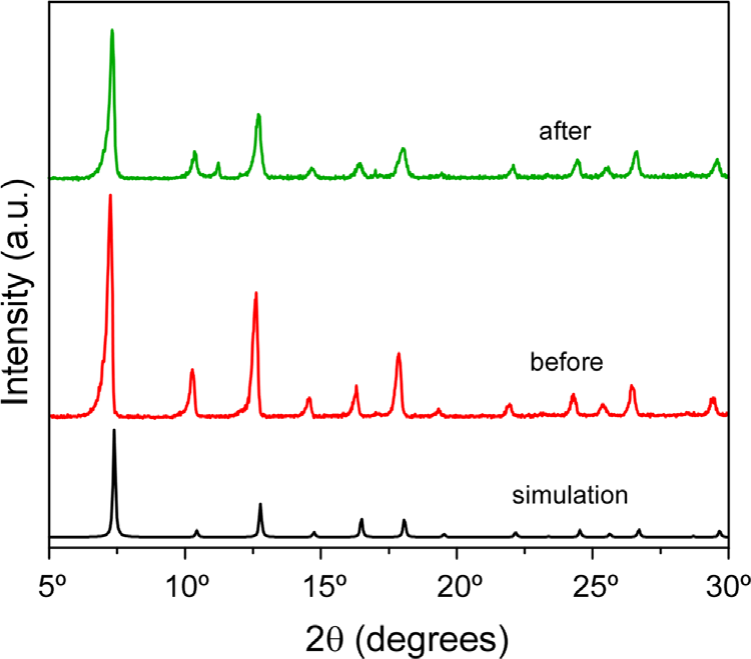
PXRD pattern of MoO_3_-NPs/ZIF-8 before and after photocatalytic reaction.

**Fig. 6:**
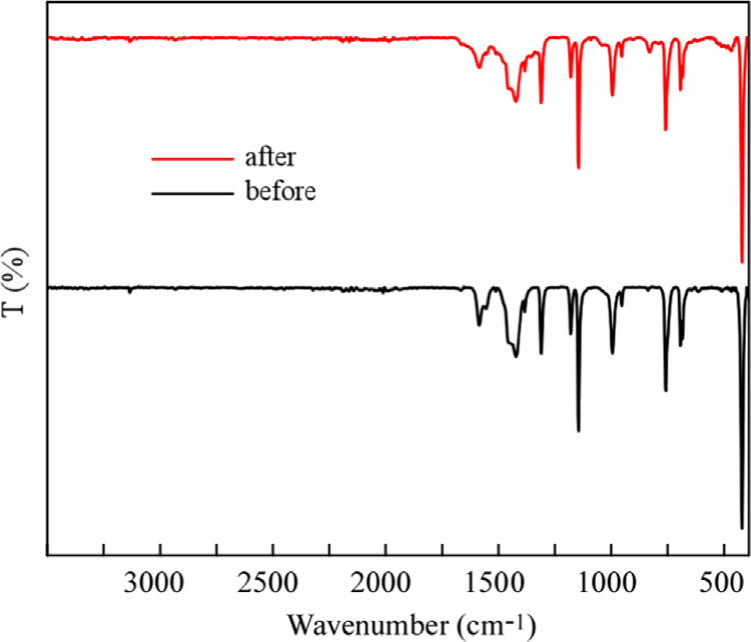
FT-IR spectra of MoO_3_-NPs/ZIF-8 before and after photocatalytic reaction.

**Fig. 7:**
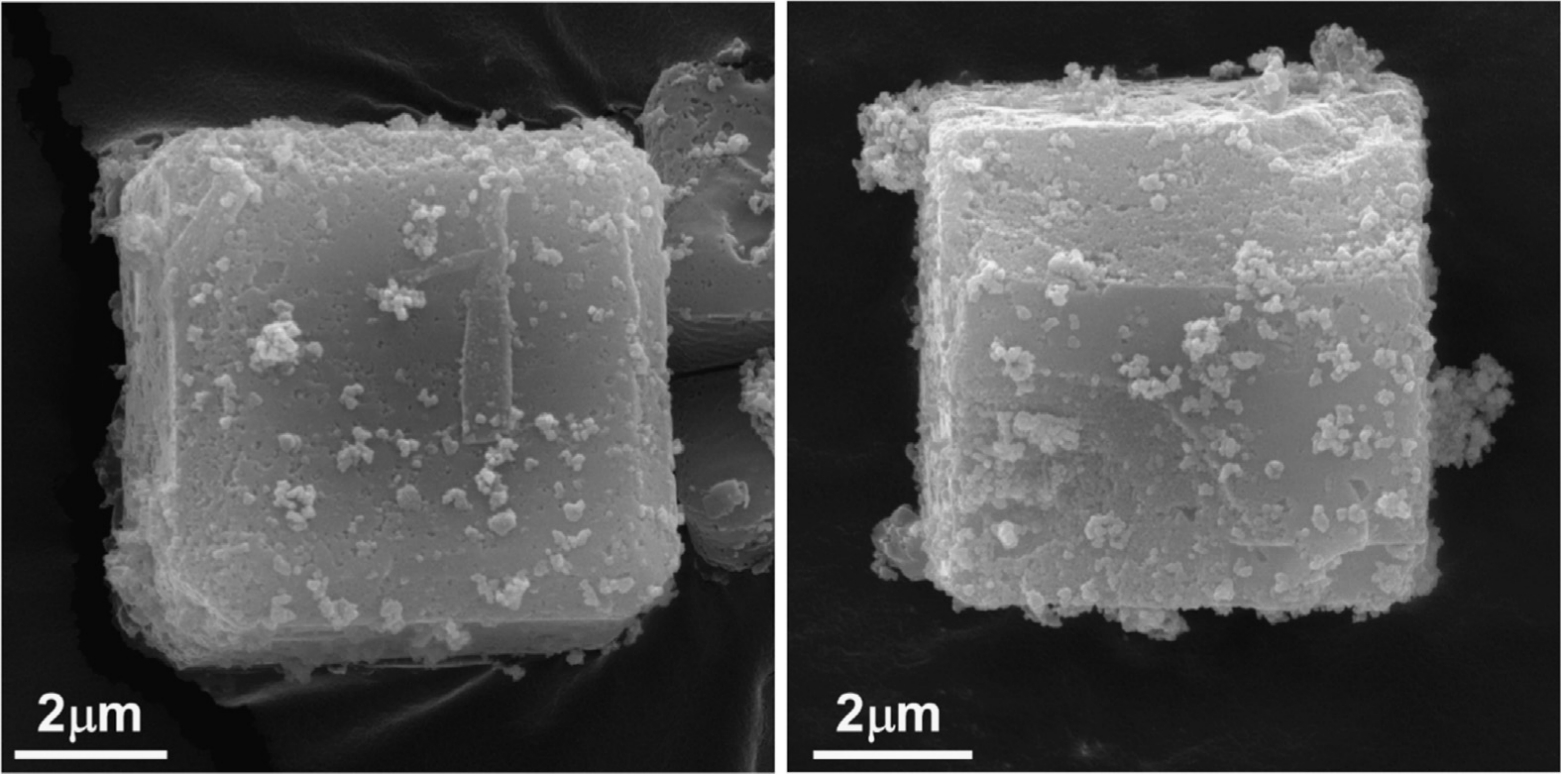
SEM pictures of MoO_3_-NPs/ZIF-8 3 wt% after use.

**Fig. 8:**
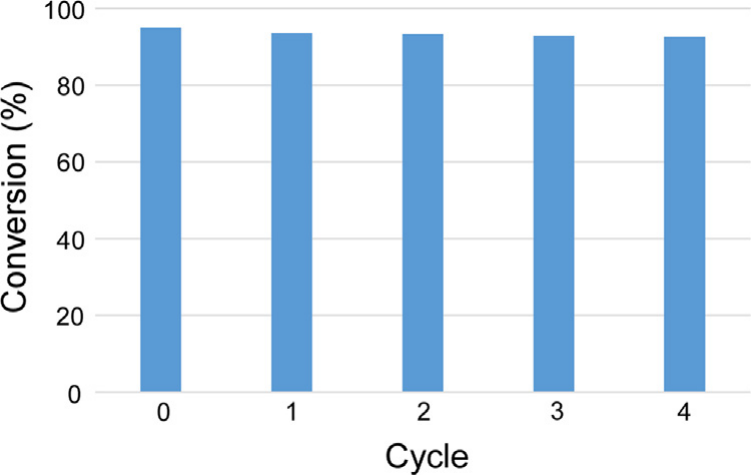
Number of catalyst recycles.

In order to assess the structural stability of the MoO_3_-NPs/ZIF-8 3 wt% after its use, the catalyst has been recycled multiple times. The PXRD spectra (Fig. 5) and FT-IR spectra (Fig. 6) reveal that the photocatalyst remains stable under the reaction condition showing negligible degradation. Furthermore, MoO_3_-NPs/ZIF-8 3 wt% morphology was characterized after the 4th cycle by SEM (Fig. 7) and the metal content was assessed by ICP-AES (Table 1). The photocatalyst has been successfully recycled four times without evident decrease in performance, as shown in Fig. 8.

**Table 1:** ICP-AES results for fresh and recycled MoO_3_-NPs/ZIF-8 3 wt%.

**MoO**_**3**_**-NPs/ZIF-8 3** **wt%**	**Metal loading (wt%)**
Fresh	3.01
Recycled	2.63

## 2 Materials, methods and experimental design

### 2.1 Materials

All reagents and solvents were purchased from commercial sources and used without further purification. The synthesis of the catalyst MoO_3_-NPs/ZIF-8 is detailed in the original paper, [*“Submitted to Microporous and Mesoporous Materials.”*] and are briefly discussed below.

### 2.2 Spray-dried ZIF-8 synthesis

The synthesis of ZIF-8 was achieved following literature procedure with minor modification [1].

Zn(OAc)_2_·2H_2_O (16 mmol) and 2-methylimidazole (16 mmol) are solubilized in 50 mL of methanol. The reaction mixture was spray-dried with a feed rate of 11.5 mL min^−1^, a flow rate of 4.6 × 10^6^ mL min^−1^ and an inlet temperature of 180 °C. The product is collected as a white powder and suspended in methanol overnight. After centrifugation the ZIF-8 is dried at 60 °C in vacuum oven.

### 2.3 ZIF-8 functionalization by rotary chemical vapor deposition

MoO_3_ nanoparticles were deposited onto ZIF-8 with the use of a rotary chemical vapor deposition (RCVD) device (Fig. 1, Fig. 2) In brief, the key feature of the RCVD is the rotary reactor chamber. Equipped with four inner blades, can ensure a sufficient contact time between the powder and the reactant gasses. A fixed amount of metal precursor [Mo(CO)_6_] is placed in the evaporator chamber at 85 °C and carried into the rotary reactor by an oxygen flow of 8.3 × 10^−7^ m^3^ s^−1^. The ZIF-8 was loaded into the rotary chamber and the deposition is set at 250 °C to prevent any degradation of the supporting material. The nanoparticle deposition process took place under reduced pressure (1.0 × 10^4^ Pa) and the deposition time (0.6 > *t* > 1.8 ks) was used to control the nanoparticles loading.

### 2.4 Photodegradation experiments

The photodegradation of methylene blue by MoO_3_-NPs/ZIF-8 was carried out as follows: 50 mg of as-synthesized catalyst were added into 200 mL dye aqueous solution (10 mg/L) and magnetically stirred for 30 minutes in a dark environment. Subsequently, the suspension is poured into a water-cooled jacked glass reactor to dissipate the intense heat sourcing from the solar lamp (PL-XQ 350 W Xenon). The solution aliquots were filtered through a 0.22 μm Millipore filter and analyzed by UV–vis absorption spectroscopy.

## 3 Data analysis

The MoO_3_-NPs/ZIF-8 photocatalytic performance is calculated based on the methylene blue photodegradation kinetics according to Eq. (1):

(1) $$-\ln(C/C_{0})\;=\;kt$$

where *C* is the MB concentration at any given time (*t*), *C*_*0*_ is the initial concentration and *k* is the rate constant.
